# Molecular characterization and genetic diversity of *Babesia bovis* and *Babesia bigemina* of cattle in Thailand

**DOI:** 10.3389/fcimb.2022.1065963

**Published:** 2022-11-29

**Authors:** Nitipon Srionrod, Pornpiroon Nooroong, Napassorn Poolsawat, Sutthida Minsakorn, Amaya Watthanadirek, Witchuta Junsiri, Siriphan Sangchuai, Runglawan Chawengkirttikul, Panat Anuracpreeda

**Affiliations:** ^1^ Parasitology Research Laboratory (PRL), Institute of Molecular Biosciences, Mahidol University, Nakhon Pathom, Thailand; ^2^ Department of Parasitology, Faculty of Medicine Siriraj Hospital, Mahidol University, Bangkok, Thailand; ^3^ Department of Microbiology, Faculty of Science, Mahidol University, Bangkok, Thailand

**Keywords:** *Babesia bovis*, *Babesia bigemina*, Babesiosis, nested PCR, genetic diversity, cattle, Thailand

## Abstract

*Babesia bovis* and *B. bigemina* are the most common tick-borne parasites that cause bovine babesiosis which effects livestock production, leading to economic losses in tropical and subtropical areas of the world. The aims of this study were to determine the molecular detection, genetic diversity and antigenicity prediction of *B. bovis* based on spherical body protein 2 (*sbp-2*) gene and *B. bigemina* based on rhoptry-associated protein 1a (*rap-1a*) gene in cattle in Thailand. By PCR assay, the molecular detection of *B. bovis* and *B. bigemina* infection revealed levels of 2.58% (4/155) and 5.80% (9/155), respectively. The phylograms showed that *B. bovis sbp-2* and *B. bigemina rap-1a* sequences displayed 5 and 3 clades with similarity ranging between 85.53 to 100% and 98.28 to 100%, respectively, when compared within Thailand strain. Diversity analysis of *sbp-2* and *rap-1a* sequences showed 18 and 4 haplotypes, respectively. The entropy analysis illustrated 104 and 7 polymorphic sites of *sbp-2* and *rap-1a* nucleic acid sequences, respectively, while those of *sbp-2* and *rap-1a* amino acid sequences showed 46 and 4 high entropy peaks, respectively. Motifs analysis exhibited the distribution and conservation among *sbp-2* and *rap-1a* sequences. The continuous and discontinuous B-cell epitopes have also been evaluated in this work. Therefore, our findings may be used to ameliorate the understanding inputs of molecular phylogeny, genetic diversity and antigenicity of *B. bovis* and *B. bigemina* Thailand stains.

## Graphical Abstract

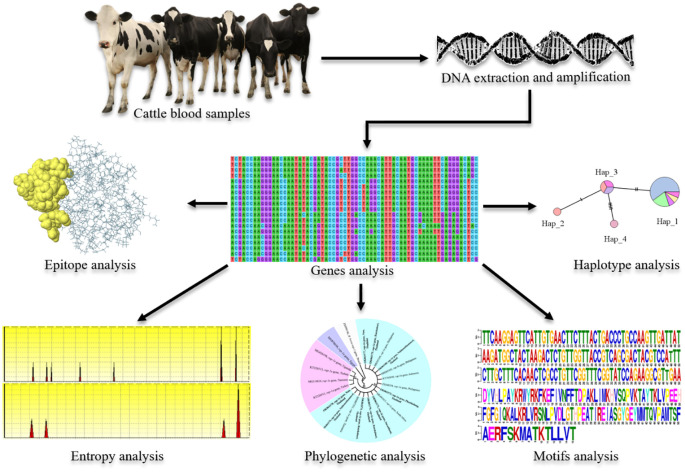

## Highlights

- The rhoptry-associated protein-1a sequences of *B. bigemina* Thailand strain exhibited a phylogenetic proximity.
- TCS network showed 18 and 4 haplotypes of the *B. bovis* spherical body protein 2 and *B. bigemina* rhoptry-associated protein-1a genes, respectively.
- Amino acid sequence entropy showed 46 and 4 polymorphic sites of the spherical body protein 2 and rhoptry-associated protein-1a, respectively.
- The *B. bovis* spherical body protein 2 sequence motif was more diverse than the *B. bigemina* rhoptry-associated protein-1a sequence motif.
- B-cell epitopes of *B. bigemina* rhoptry-associated protein-1a sequences were conserved, while that of *B. bovis* spherical body protein 2 sequences were diverse.

## Introduction

Bovine babesiosis is an important tick-borne hemoparasitic disease caused by *Babesia bovis* and *B. bigemina*, and transmitted by ticks (Silva et al., 2009; Sayed and Elshahawy, 2018). This disease leads to economic losses in livestock production in tropical and subtropical areas including Thailand (Uilenberg, 2006; Yusuf, 2017), with estimated losses of US$13.9-18.7 billion globally each year (Hurtado and Giraldo-Ríos, 2018; Marques et al., 2020). *B. bovis* and *B. bigemina* cause morbidity and mortality resulting in losses in milk and meat production, and other livestock by-products. Although *B. bigemina* is more widespread, *B. bovis* infection is the most critical and fatal because of its clinical manifestations including hyperthermia, anorexia, hemoglobinuria and neurological symptoms (Bock et al., 2004; Uilenberg, 2006; Sayed and Elshahawy, 2018). The diagnostic methods of bovine babesiosis have relied mostly on clinical signs (Wagner et al., 2002; Sayed and Elshahawy, 2018), microscopic examination of the parasites in Giemsa-stained blood smears (Almeria et al., 2001; Bock et al., 2004; Kumar et al., 2018; Mendes et al., 2019) and serological testing for antibody detection (Bono et al., 2008; Singh et al., 2009; Li et al., 2014; Lira-Amaya et al., 2021). The molecular method by polymerase chain reaction (PCR) is reliable and employed in diagnosing the infection (Kumar et al., 2018; Mendes et al., 2019). The occurrence of bovine babesiosis in different parts of Thailand has been reported in previous studies (Cao et al., 2012; Nagano et al., 2013; Simking et al., 2014; Tattiyapong et al., 2016; Jirapattharasate et al., 2016; Jirapattharasate et al., 2017).

The spherical body proteins (SBPs) secreted by spherical bodies are identified to belong to a family consisting of *sbp-1*, *sbp-2*, *sbp-3* and *sbp-4*, which have been characterized in *B. bovis* (Yokoyama et al., 2006; Guo et al., 2018). The *B. bovis sbp-2* protein is encoded by *sbp-2* gene and has been identified as an immunostimulatory protein released into the host erythrocyte post-invasion and localized to the cytoplasmic side of the infected-erythrocyte membrane (Ruef et al., 2000; Yokoyama et al., 2006; Terkawi et al., 2011b; Lobo et al., 2012; Ganzinelli et al., 2018). The rhoptry-associated protein-1a is secreted by rhoptries organelles that participate in the success of invasion and establishment of intracellular parasitic viability (Cao et al., 2012; Kemp et al., 2013). The *B. bigemina rap-1a* gene is conserved and used for discriminating against field *B. bigemina* (Petrigh et al., 2008; Cao et al., 2012). Thus, both of *B. bovis sbp-2* and *B. bigemina rap-1a* genes were candidates for molecular markers and potent in identifying specific *B. bovis* and *B. bigemina*, respectively (AbouLaila et al., 2010; Moumouni et al., 2015).

The scarcity of the genotyping and epidemiology information which might be used to control the disease is not available. And this includes the fact that little is known regarding the genetic diversity of *B. bovis* and *B. bigemina* isolates in Thailand (Sivakumar et al., 2018). Therefore, this study was done with the main objective of investigating molecular detection and genetic diversity of *B. bovis sbp-2* and *B. bigemina rap-1a* genes in cattle from Northern, Western and Southern regions of Thailand. Additionally, *in silico* bioinformatic analyses among the isolated sequences identified in this work and those from other countries are presented. The results will provide further information on the genetic structure of these parasite populations.

## Materials and methods

### Collection of blood samples

The present study was carried out in three areas of Thailand. A total of 155, blood samples from apparently healthy cattle (Holstein Friesian cross-bred) aged between 1 and 5 years were randomly collected in Muang district (18°44′22″ N, 100°41′4″ E) of Nan province (n = 50) in northern area, in Photharam district (13°44′20″ N, 99°55′25″ E) of Ratchaburi province (n = 54) in western area as well as in Don-sak district (9°7′8″ N, 99°42′20″ E) of Surat Thani province (n = 15), Cha-wang district (8°28′10″ N, 99°32′3″ E) of Nakhon Si Thammarat province (n = 16) and Pa-phayom district (7°48′11″ N, 99°55′29″ E) of Phatthalung province (n = 20) in Southern area. Approximately 5 ml of whole blood samples were collected directly from the middle caudal coccygeal vein of each animal. They were transferred to a collection tube with Ethylene Diamine Tetraacetic Acid (EDTA) (BD Vacutainer^®^, USA), and stored at -20°C until the DNA extraction took place. The procedures of animal restraint and blood collection were performed by a licensed veterinarian.

### DNA extraction

Genomic DNA (gDNA) of *B. bovis* and *B. bigemina* was extracted from cattle blood samples using a Tissue DNA Extraction Kit (OMEGA, bio-tex, USA) following the methods described by Junsiri et al. (2020) and Watthanadirek et al. (2021) with some modifications. Briefly, 250 µl of blood sample was transferred into a 1.5 ml microcentrifuge tube, and 25 µl of Omega Bio-tek (OB) protease solution and 250 µl of BL buffer (lysis buffer) were added. They were mixed and incubated for 10 min at 70^°^C. Then, 250 µl of absolute ethanol was added, and the sample was transferred to a HiBind^®^ DNA Mini Column (OMEGA, bio-tex, USA), and centrifuged at maximum speed for 1 min at room temperature. After removing the filtrate, 500 µl of HBC binding buffer (High Salt Wash Buffer) was added and centrifuged at maximum speed for 30 sec at room temperature. After discarding the filtrate, 700 µl of DNA washing buffer was added and centrifuged at maximum speed for 30 sec at room temperature. Then, the column was transferred to a new microcentrifuge tube, and the DNA samples were eluted in 50 µl of MiliQ water. Finally, the purity and concentration of DNA samples were determined using NanoDrop™ 2000 Spectrophotometers (Thermo Scientific™) at the 260/280 and 260/230 ratios, and stored at -20^°^C until further use.

### Molecular amplification and cloning of the *Babesia bovis sbp-2* gene and *Babesia bigemina rap-1a* gene DNA

All specific primer pairs according to sequences from the previous studies (AbouLaila et al., 2010; Moumouni et al., 2015) were used for amplification of the *B. bovis sbp-2* and *B. bigemina rap-1a* genes. The *B. bovis sbp-2* and *B. bigemina rap-1a* genes were amplified by nested PCR assay using the specific primers. In the first step of amplification, outer forward F (5′-CTGGAAGTGGATCTCATGCAACC-3′) and outer reverse R (5′-TCACGAGCACTCTACGGCTTTGCAG-3′) were used for *B. bovis sbp-2* gene, while outer forward F (5′-GAGTCTGCCAAATCCTTAC-3′) and outer reverse R (5′-TCCTCTACAGCTGCTTCG-3′) were used for *B. bigemina rap-1a* gene. In the second step, 2 µl (350 ng/µl) of the amplified PCR product was used for the subsequent nested PCR. The specific primers: inner forward F1 (5′-GAATCTAGGCATATAAGGCAT-3′) and inner reverse R1 (5′-CCCCTCCTAAGGTTGGCTAC-3′) were used for *B. bovis sbp-2* gene, while inner forward F2 (5′-AGCTTGCTTTCACAACTCGCC-3′) and inner reverse R2 (5′-TTGGTGCTTTGACCGACGACAT-3′) were used for *B. bigemina rap-1a* gene. The PCR reaction containing 50 ng of DNA template, 0.2 µM each of the primers, 200 µM of each deoxynucleotide triphosphate (dNTPs), 1x standard Taq reaction buffer, nuclease-free water and 1.25 U Taq DNA polymerase (BioLabs^®^, USA), were performed in a thermal cycler (Bio-Rad, USA) with the following conditions: 35 cycles of denaturation at 94^°^C for 2 min, annealing at 60^°^C and 50^°^C for 1 min for the 1^st^ and 2 ^nd^ steps of *B. bovis sbp-2* gene, at 47^°^C and 56^°^C for 30 sec for 1^st^ and 2 ^nd^ steps of *B. bigemina rap-1a* gene, extension at 68^°^C for 90 sec, and a final extension at 68^°^C for 5 min. The PCR products were stained with FluoroStain™ DNA Fluorescent Staining Dye (SMOBIO, Taiwan), analyzed by 1% agarose gels and visualized under ultraviolet (UV) transilluminator. A 100 bp DNA Ladder M (MolBio™ HIMEDIA^®^, India) was used as standard for determining the molecular mass of PCR products. Positive PCR products were purified using GenepHlow™ Gel/PCR Kit (Geneaid, Taiwan) following the manufacturer’s protocols for DNA cloning. The purified PCR products were quantified and cloned into the pGEM^®^-T Easy Vector (Promega, USA). Chemically competent *Escherichia coli* host strain TOP10 cells (Invitrogen, USA) were then transformed with the ligation products. Subsequently, 200 µl of transformed *E. coli* culture was spread on the Luria-Bertani (LB)-ampicillin agar plates after 100µl of 100mM IPTG, and 20µl of 50mg/ml X-Gal were spread on LB-ampicillin plate, then the plates were incubated at 37°C for overnight. The white colonies were selected and grown in LB medium containing ampicillin for overnight. The recombinant plasmids were extracted from the competent cells using Presto™ Mini Plasmid Kit (Geneaid, Taiwan) following the manufacturer’s instructions. The plasmid DNA products were amplified using the specific primers and the correct size of the inserts was analyzed by agarose gel electrophoresis.

### Sequence and *in silico* analysis

Purified plasmid DNA products containing the *B. bovis sbp-2* and *B. bigemina rap-1a* genes were confirmed by the Sanger method of DNA sequencing. All sequences were analyzed by BLAST (The National Center for Biotechnology Information, NCBI, http://www.ncbi.nlm.nih.gov/BLAST). All DNA sequences generated in the present study were submitted and deposited in GenBank database; accession numbers are provided in **Table 1**.

**Table 1:** The *Babesia* spp. nucleotide sequences amplified in Thailand strain were deposited in the GenBank database.

Regions	Provinces	Districts	Animal ID	GenBank accession numbers	
				*sbp-2 B. bovis* gene	*rap-1a B. bigemina* gene
Northern	Nan	Muang	NAN2	OK061184	
			NAN4		OK061177
			NAN39		OK061178
			NAN49	OK061185	
Western	Ratchaburi	Photharam	POA1		OK061174
			POC4		OK061175
			BPA1		OK061176
Southern	Surat Thani	Don-sak	DSSK5		OK061172
			DSSK8	OK061181	
	Nakhon Si Thammarat	Cha-wang	CW8		OK061179
			CW16		OK061180
	Phatthalung	Pa-phayom	PLT1		OK061173
			PLT8	OK061182	

### Phylogenetic tree analysis

The *B. bovis sbp-2* and *B. bigemina rap-1a* gene sequences were utilized for sequence alignment and phylogenetic analysis. Multiple sequences alignments were carried out with the Clustal W algorithm, and then molecular phylogenetic trees were reconstructed using the maximum likelihood (ML) as implemented in the MEGA software v.7.0.26 (Kumar et al., 2016). Bootstrap analysis with 1000 repetitions was used to evaluate the confidence of the branching pattern of the trees. The evolutionary distances were computed using the Kimura 2-parameter method (Kimura, 1980). The *sbp-4* and *rap-1* sequences of *B. bovis* were used as appropriate outgroups for the construction of the phylogenetic trees of *B. bovis sbp-2* and *B. bigemina rap-1a* sequences, respectively. The similarity of nucleic acid was also defined with a sequence identity matrix in Bioedit software v.7.0.5.3 (Hall, 1999).

### Haplotype diversity analysis

The alignment of *B. bovis sbp-2* and *B. bigemina rap-1a* sequences were used to assess the nucleotide diversity (π), diversity of haplotypes (Dh), number of haplotypes, and average number of nucleotide differences (*K*), using DNA Sequence Polymorphism (DnaSP) version 6.12.03 software (Rozas et al., 2017). In addition, all sequences were subjected to the Population Analysis with the Reticulate Trees (popART) program in order to analyze the Templeton, Crandall, and Sing (TCS) Network (Clement et al., 2002; Leigh and Bryant, 2015).

### Entropy and motif analysis

The variability between the nucleic and amino acid sequences was analyzed by entropy estimation. The *B. bovis sbp-2* and *B. bigemina rap-1a* nucleotide sequences were converted to amino acid sequences, aligned and analyzed by Shannon’s entropy (H(x)) plot using Bioedit software version 7.2.5 (Hall, 1999). Also, the motif analysis of nucleic and amino acid was performed in multiple expectation maximizations for motif elicitation (MEME) suite tools (https://meme-suite.org/meme/) with E-values less than 0.05 (Bailey and Elkan, 1994).

### B-cell epitopes prediction

Nucleotide sequences of *B. bovis sbp-2* and *B. bigemina rap-1a* genes were translated to amino acid sequences and then submitted to the VaxiJen v.2.0 Server and the Immune Epitope Database and Analysis Resource (IEDB-AR, http://tools.iedb.org/bcell/) for B-cell epitopes analysis (Vita et al., 2019). The antigenic determinants on protein antigens were predicted by a semi-empirical method (Kolaskar and Tongaonkar, 1990). Linear sequences-based analysis of the *B. bovis sbp-2* and *B. bigemina rap-1a* was used to predict the continuous B-cell epitopes, BepiPred linear epitope, Parker hydrophilicity, Emini surface accessibility, Chou & Fasman Beta-turn and Karplus & Schulz flexibility. The 3D structure of antigen was evaluated using the Ellipro Server for prediction of the discontinuous B-cell epitopes

## Results

### Molecular detection of *Babesia* infection in cattle blood samples

The *Babesia* spp. detected by nested PCR was demonstrated, and the size of PCR products of *B. bovis* and *B. bigemina* were 584 and 412 bp, respectively. The representative positive PCR products for each of the parasites tested were exhibited in **Supplementary Figure 1**. The PCR results showed that 4/155 (2.58%) and 9/155 (5.80%) of cattle blood samples were infected with *B. bovis* and *B. bigemina*, respectively. *B. bovis* infections were found in Nan, Surat Thani and Phatthalung provinces, whilst *B. bigemina* infections were mainly detected in Ratchaburi, followed by Nan, Surat Thani, Nakhon Si Thammarat and Phatthalung provinces as shown in **Table 1**.

### Phylogenetic, similarity and sequence analysis of the *Babesia bovis sbp-2* and *Babesia bigemina rap-1a* gene sequences

**Figure 1:**
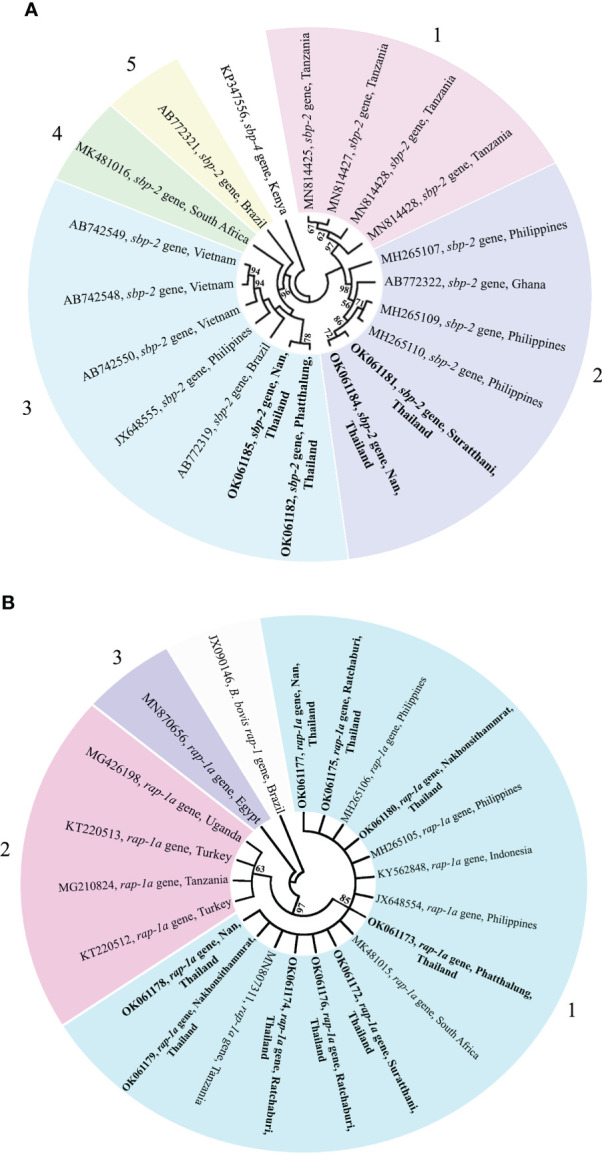
A maximum likelihood phylogenetic cladogram of *B bovis sbp-2* **(A)** and *B bigemina rap-1a* **(B)** gene sequences in this study (boldface) and those taken from GenBank. The numbers on each node are consistent with the bootstrap analysis of 1000 replicates (only percentage greater than 60% were represented). The GenBank accession numbers of the sequences used in the phylogenetic analysis are exhibited. Two sequences of *B bovis sbp-4* **(A)** and *rap-1* **(B)** gene are used as outer groups. Different colors represent the different clades.

The phylogenetic tree based on the sequence alignment of the *B. bovis sbp-2* gene obtained in this study along with the other sequences retrieved from the GenBank was divided into 5 clades (designated as clade 1-5). The Thailand sequences are positioned in the 2^nd^ and 3^rd^ clade together with the sequences from other countries (**Figure 1A**) The percentage similarity of Thailand *B. bovis* sequences within each clade was 85.53-100% (2^nd^ clade) and 95.40-100% (3^rd^ clade), while the similarity of the sequences within other clades was 85.57-100% (1^st^ clade), 89.07-100% (4^th^ clade) and 100% (5^th^ clade) as shown in **Table 2**. For phylogenetic tree of the *B. bigemina rap-1a* gene, the sequences were classified as 3 clades in the phylogram. Our nine sequences were found in clade 1 together with the sequences from Philippines, Indonesia and South Africa. The Thailand sequences showed phylogenetic proximity representing the genetic variability of *B. bigemina rap-1a* sequence from this study (**Figure 1B**). The total similarity among Thailand *B. bigemina* sequences was 100% (1^st^ clade), whereas the similarity of the remaining sequences within other clades was 98.53-100% (2^nd^ clade) and 100% (3^rd^ clade) as demonstrated in **Table 3**.

**Table 2:** Similarity of the *B. bovis sbp-2* gene sequences as examined in cattle samples in Thailand and other countries.

Clade	1				2						3							4	5
Accesion number	1	2	3	4	5	6	7	8	9	10	11	12	13	14	15	16	17	18	19
1. MN814425	100.00																		
2. MN814427	100.00	100.00																	
3. MN814428	99.66	99.66	100.00																
4. MG725962	99.66	99.66	99.31	100.00															
5. MH265107	91.63	91.63	91.44	92.02	100.00														
6. AB772322	91.43	91.43	91.25	91.82	94.47	100.00													
7. MH265109	92.02	92.02	91.83	92.40	95.95	96.30	100.00												
8. MH265110	92.02	92.02	91.83	92.40	95.58	96.30	99.66	100.00											
9. OK061181	89.66	89.66	89.47	90.06	94.11	94.29	97.20	96.84	100.00										
10. OK061184	91.22	91.22	91.04	91.62	96.12	95.57	98.79	98.44	97.20	100.00									
11. OK061182	85.98	85.98	85.57	86.41	87.85	87.21	87.64	87.22	85.53	86.36	100.00								
12. OK061182	89.06	89.06	88.66	89.46	89.28	89.45	88.66	88.66	86.37	87.82	95.40	100.00							
13. AB772319	87.03	87.03	86.63	87.45	87.27	85.60	86.84	86.42	84.93	86.62	91.28	90.89	100.00						
14. JX648555	89.43	89.43	89.04	89.83	88.42	86.95	88.00	87.58	85.49	87.38	92.58	93.90	93.72	100.00					
15. AB742550	89.46	89.46	89.06	89.86	87.86	87.62	88.05	88.05	85.74	87.20	91.85	94.11	93.75	96.11	100.00				
16. AB742548	89.27	89.27	88.88	89.68	88.47	88.24	88.66	88.66	86.37	87.82	91.66	93.56	93.56	95.56	98.79	100.00			
17. AB742549	89.07	89.07	88.68	89.48	88.27	88.03	88.45	88.45	86.15	87.82	91.85	93.37	93.56	95.38	98.62	99.14	100.00		
18. MK481016	90.24	90.24	89.85	89.84	88.04	87.84	89.46	89.05	87.43	88.65	92.77	91.62	92.02	92.37	92.59	92.78	92.59	100.00	
19. AB772321	91.03	91.03	90.85	91.43	89.08	90.28	89.89	89.89	88.27	89.07	87.64	90.43	86.44	88.97	89.45	89.28	89.07	89.83	100.00
	Similarity (%)																		

**Table 3:** Similarity of the *B. bigemina rap-1a* gene sequences as detected in cattle samples in Thailand and other countries.

Clade	1															2				3
Accesion number	1	2	3	4	5	6	7	8	9	10	11	12	13	14	15	16	17	18	19	20
1. OK061177	100.00																			
2. OK061175	100.00	100.00																		
3. MH265106	100.00	100.00	100.00																	
4. OK061180	100.00	100.00	100.00	100.00																
5. MH265105	100.00	100.00	100.00	100.00	100.00															
6. KY562848	100.00	100.00	100.00	100.00	100.00	100.00														
7. JX648554	100.00	100.00	100.00	100.00	100.00	100.00	100.00													
8. OK061173	100.00	100.00	100.00	100.00	100.00	100.00	100.00	100.00												
9. MK481015	100.00	100.00	100.00	100.00	100.00	100.00	100.00	100.00	100.00											
10. OK061172	100.00	100.00	100.00	100.00	100.00	100.00	100.00	100.00	100.00	100.00										
11. OK061176	100.00	100.00	100.00	100.00	100.00	100.00	100.00	100.00	100.00	100.00	100.00									
12. OK061174	100.00	100.00	100.00	100.00	100.00	100.00	100.00	100.00	100.00	100.00	100.00	100.00								
13. MN807311	100.00	100.00	100.00	100.00	100.00	100.00	100.00	100.00	100.00	100.00	100.00	100.00	100.00							
14. OK061179	100.00	100.00	100.00	100.00	100.00	100.00	100.00	100.00	100.00	100.00	100.00	100.00	100.00	100.00						
15. OK061178	100.00	100.00	100.00	100.00	100.00	100.00	100.00	100.00	100.00	100.00	100.00	100.00	100.00	100.00	100.00					
16. KT220513	99.27	99.27	99.27	99.27	99.27	99.27	99.27	99.27	99.27	99.27	99.27	99.27	99.27	99.27	99.27	100.00				
17. MG210824	99.51	99.51	99.51	99.51	99.51	99.51	99.51	99.51	99.51	99.51	99.51	99.51	99.51	99.51	99.51	99.76	100.00			
18. KT220512	99.51	99.51	99.51	99.51	99.51	99.51	99.51	99.51	99.51	99.51	99.51	99.51	99.51	99.51	99.51	99.76	100.00	100.00		
19. MG426198	99.51	99.51	99.51	99.51	99.51	99.51	99.51	99.51	99.51	99.51	99.51	99.51	99.51	99.51	99.51	99.76	100.00	100.00	100.00	
20. MN870656	98.28	98.28	98.28	98.28	98.28	98.28	98.28	98.28	98.28	98.28	98.28	98.28	98.28	98.28	98.28	98.53	98.78	98.78	98.78	100.00
	Similarity (%)																			

In addition, the nucleic acid substitution rate in *sbp-2* sequences among *B. bovis* and *rap-1a* sequences among *B. bigemina* was evaluated under the Tamura and Nei (1993) mode. The different transitional and transversional substitutions of *B. bovis sbp-2* sequences were 14.06 and 5.47, respectively, while those of *B. bigemina rap-1a* sequences were 18.79 and 3.11, respectively (**Supplementary Table 1**). The alignment of *B. bovis sbp-2* sequence of Thailand strains and other strains revealed the 420 and 162 positions of consensus and non-consensus, respectively. The consensus and non-consensus of *B. bigemina rap-1a* sequences alignment were 404 and 8 positions, respectively, as demonstrated in **Supplementary Figures 2** and **3**. In addition, the consensus and non-consensus of *B. bovis sbp-2* amino acid sequence alignments were 121 and 72 positions, respectively, while the alignment of *B. bigemina rap-1a* amino acid sequences revealed 132 and 4 positions of the consensus and non-consensus, respectively (**Supplementary Figure 4**). Seventy-two positions of the amino acid (aa) sequences revealed high genetic variation among *B. bovis* strains (**Supplementary Table 2**), whereas four aa were found in the genetic variation among *B. bigemina* strains (**Supplementary Table 3**). The highest variation of *rap-1a* aa position among 20 sequences was aa 130 [from Asparagine (Asn; N) to Aspartic acid (Asp; D)], as shown in **Supplementary Table 3**.

### Haplotype diversity

**Figure 2:**
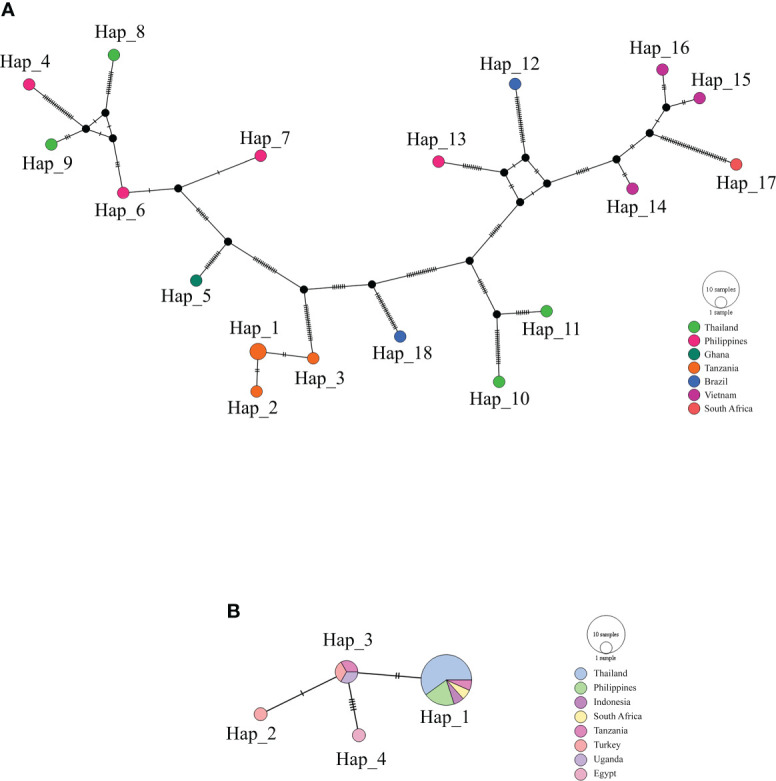
TCS network of haplotypes based on the *B bovis sbp-2* **(A)** and *B bigemina rap-1a* **(B)** gene sequences detected in Thailand and worldwide countries. Small traits between a haplotype and another stand for mutational occurrence. The black circles are the intermediated traits caused by the single nucleotide polymorphism (SNP).

The haplotype network of *B. bovis sbp-2* and *B. bigemina rap-1a* gene sequences was established by the TCS Network tool. The haplotype of each gene was estimated and displayed high variation from multiple sequence alignments. The *B. bovis sbp-2 gene* showed a greater number of nucleotide variations and higher diversity than *B. bigemina rap-1a* gene as demonstrated in **Table 4** and **Figure 2**. For haplotype analysis of *B. bovis sbp-2* gene, 18 haplotypes shown in TCS network displayed that haplotype #9 and #11 were obtained from Nan province, haplotype #8 was found in Surat Thani province, and haplotype #10 was observed from Phatthalung province of Thailand. Our haplotypes from Thailand formed the nearest branch to the haplotype from the Philippines. The rest of the haplotypes from the Philippines, Ghana, Tanzania, Brazil, Vietnam, South Africa were exhibited in **Figure 2A**. In addition, a total of 4 different haplotypes were analyzed based on the *B. bigemina rap-1a* gene. Our nine sequences from Nan, Ratchaburi, Surat Thani, Nakhon Si Thammarat and Phatthalung provinces of Thailand were grouped together in haplotype #1 with the Philippines, Tanzania, South Africa, Indonesia. The rest of the haplotypes were obtained from other countries as shown in **Figure 2B**.

**Table 4:** Polymorphism and genetic diversity of the *B. bovis sbp-2* and *B. bigemina rap-1a* gene sequences as examined in cattle samples in Thailand and other countries.

Genes	Size (bp)	N	VS	GC%	h	Dh (mean ± SD)	π (mean ± SD)	*K*
**Sequence with this study* ***								
*sbp-2*	581	4	90	44.6	4	1.000 ± 0.177	0.09348 ± 0.02221	54.50000
*rap-1a*	412	9	0	52.4	NP	NP	0.000 ± 0.000	NP
**Sequence worldwide**								
*sbp-2*	581	19	162	44.3	18	0.994 ± 0.019	0.08566 ± 0.00426	49.76608
*rap-1a*	412	20	8	52.5	4	0.432 ± 0.126	0.00337 ± 0.00137	1.38947

N, number of analyzed sequence; VS, number of variable sites; GC, G x C content; h, number of haplotypes;

Dh, diversity of haplotypes; SD, standard deviation; π, nucleotide diversity (per site);

K, average number of nucleotide differences; NP, No polymorphism.

### Entropy and motif analyses

**Figure 3:**
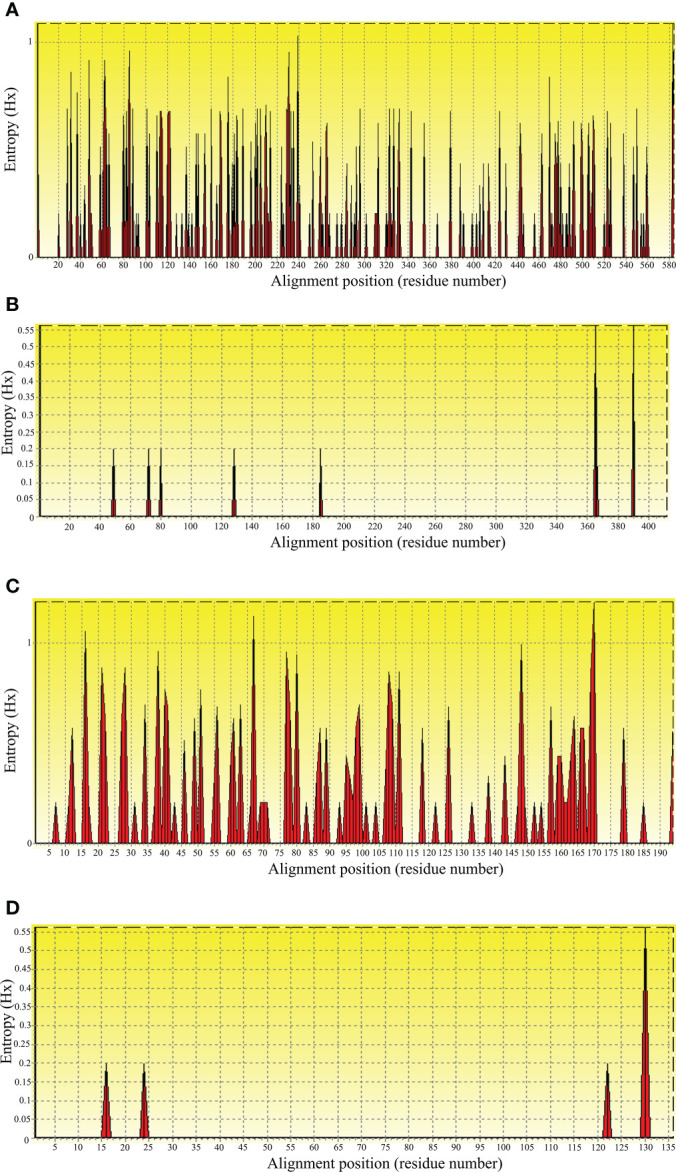
Entropy analysis of *Babesia* gene sequences. Entropy plot of multiple nucleic acid sequence alignment of *B bovis sbp-2* **(A)** and *B bigemina rap-1a* **(B)** genes. Entropy plot of multiple amino acid sequence alignment of *sbp-2* **(C)** and *rap-1a* **(D)**. The red peaks indicate the high variation at each position of the nucleic **(A, B)** and amino **(C, D)** acid sequences.

To evaluate nucleic acid entropy, the *B. bovis sbp-2* gene sequences showed 104 polymorphic sites with entropy values ranging from 0.20619 to 1.02809 (**Figure 3A**), while the *B. bigemina rap-1a* gene sequences exhibited 7 polymorphic sites with entropy values ranged between 0.19852 and 0.56234 (**Figure 3B**). Entropy analysis of amino acid sequences was conducted using the *sbp-2* and *rap-1a* amino acid sequences alignments. The charts exhibited 46 and 4 high entropy peaks for the *sbp-2* (**Figure 3C**) and *rap-1a* (**Figure 3D**) amino acid sequences, respectively. The entropy values were observed with the range of 0.20619 to 1.2064 for *sbp-2* (**Figure 3C**) and 0.19852 to 0.56234 for *rap-1a* (**Figure 3D**) amino acid sequences, respectively.

**Figure 4:**
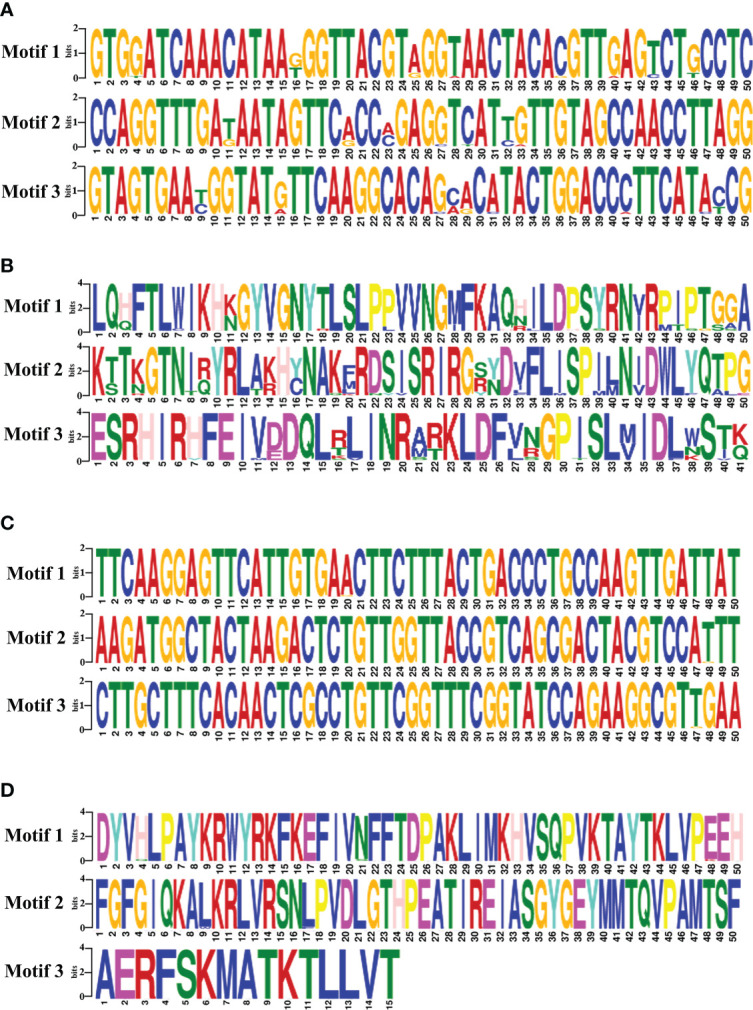
The motifs analysis of *B bovis sbp-2* nucleic **(A)** and amino **(B)** acid sequences as well as *B bigemina rap-1a* nucleic **(C)** and amino **(D)** acid sequences.

The motifs analysis of *B. bovis sbp-2* and *B. bigemina rap-1a* gene sequences showed three conserved motifs in each sequence. These motifs are distributed and conserved among the *sbp-2* and *rap-1a* gene sequences. Although the DNA motif was highly conserved, the *sbp-2* DNA motif was more diverse than the *rap-1a* DNA motif (**Figures 4A, C**). Also, the motif analysis of the *sbp-2* and *rap-1a* amino acid sequences exhibited three conserved motifs among each amino acid sequence (**Figures 4B, D**). Our motif analysis results were consistent with other results including the genetic diversity, similarity, haplotype, entropy and the sequence alignment, which is the *B. bovis sbp-2* has more diversity than *B. bigemina rap-1a* in both of DNA and amino acid sequences.

### B-cell epitopes analysis

**Figure 5:**
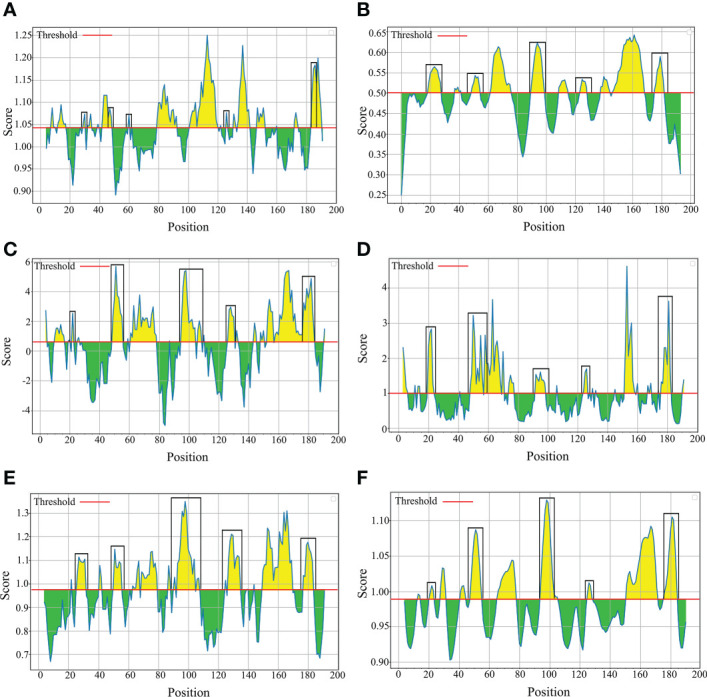
The physicochemical properties of *B bovis sbp-2* protein were assessed based on Kolaskar & Tongaonkar antigenicity **(A)**, BepiPred linear epitope **(B)**, Parker hydrophilicity **(C)**, Emini surface accessibility **(D)**, Chou & Fasman Beta-turn **(E)** and Karplus & Schulz flexibility prediction **(F)**.

**Figure 6:**
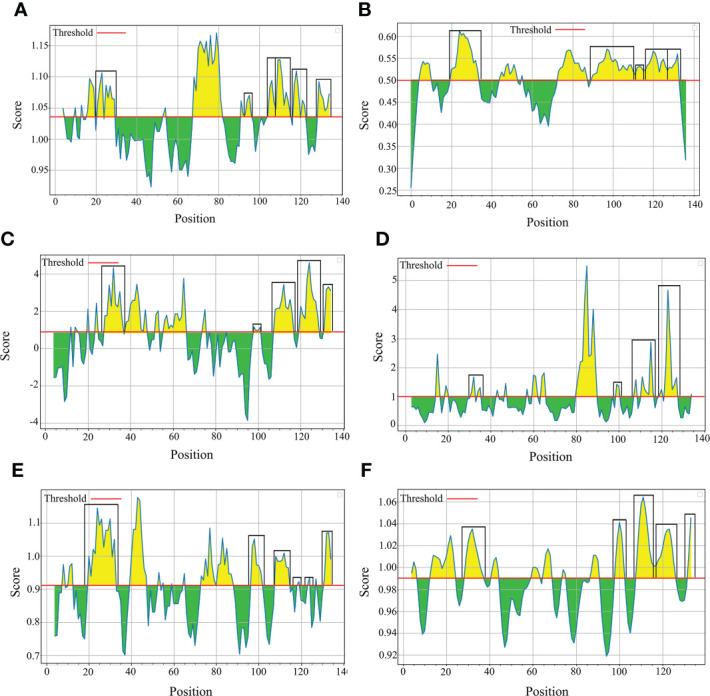
The physicochemical properties of *B bigemina rap-1a* protein were assessed based on Kolaskar & Tongaonkar antigenicity **(A)**, BepiPred linear epitope **(B)**, Parker hydrophilicity **(C)**, Emini surface accessibility **(D)**, Chou & Fasman Beta-turn **(E)** and Karplus & Schulz flexibility prediction **(F)**.

**Figure 7:**
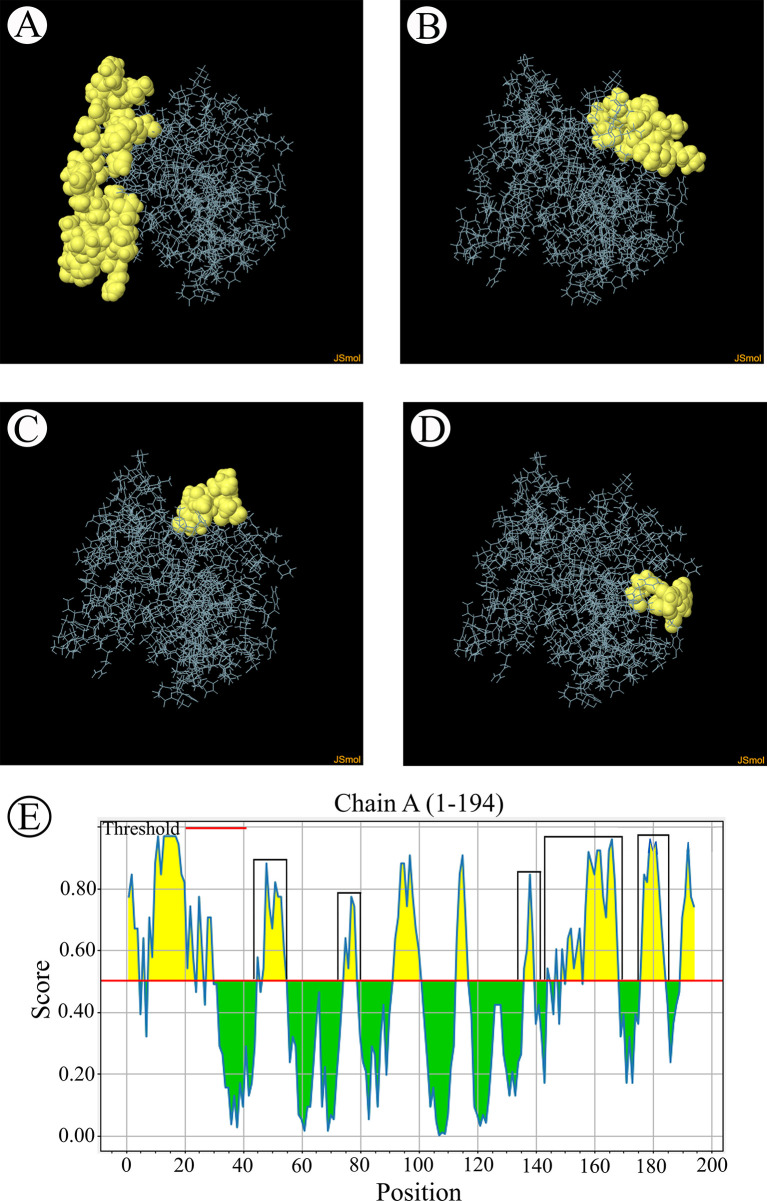
The predicted discontinuous B-cell epitopes based on the 3D structure of *B bovis sbp-2* protein **(A–D)** displayed the overlapping region in the same position as the B-cell linear structure prediction **(E)**.

**Figure 8:**
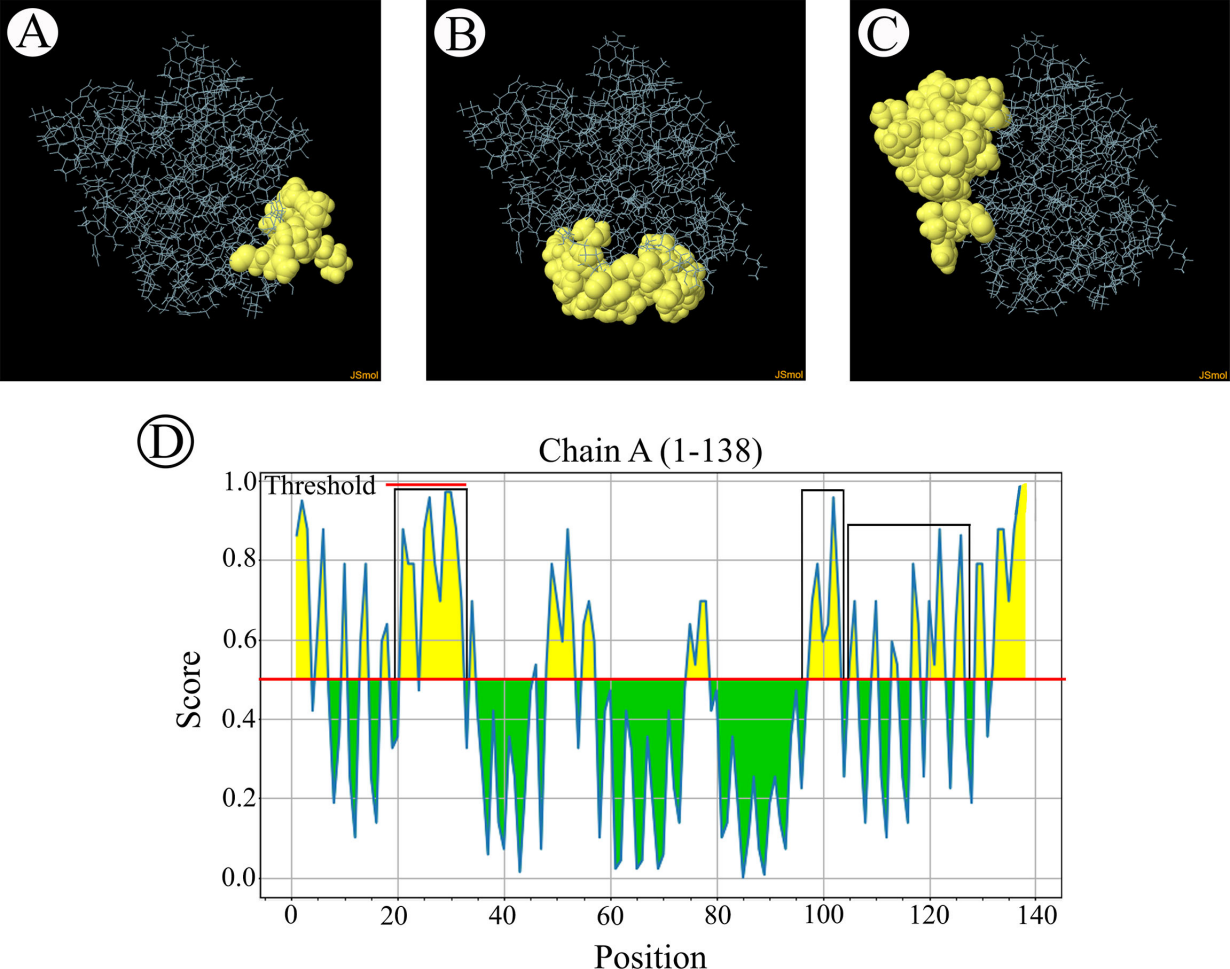
The predicted discontinuous B-cell epitopes based on the 3D structure of *B bigemina rap-1a* protein **(A–C)** displayed the overlapping region in the same position as the B-cell linear structure prediction **(D)**.

The B-cell epitopes predicted among Thailand *B. bovis sbp-2* and *B. bigemina rap-1a* amino acid sequences were conserved, and all others were observed to be highly polymorphic (**Table 5**). The continuous B-cell epitopes were anticipated for antigenic properties along with amino acid sequences by the Kolaskar & Tongaonkar antigenicity algorithm. The average antigenicity of *sbp-2* (**Figure 5A**) and *rap-1a* (**Figure 6A**) amino acid sequences were 1.041 (minimum = 0.891; maximum = 1.250) and 1.035 (minimum = 0.923; maximum = 1.170), respectively. In addition, the physicochemical properties of continuous B-cell epitopes were analyzed by BepiPred linear epitope, Chou & Fasman Beta-turn, Emini surface accessibility, Karplus & Schulz flexibility and Parker hydrophilicity predictions. Our findings exhibited an overlapping amino acid region that could be a candidate for B-cell epitopes recognition and accessible for antibody binding (**Figures 5B–F** and **6B–F**). However, the discontinuous B-cell epitopes were predicted based on the 3D structure of *sbp-2* (**Supplementary Table 4** and **Figures 7A–D**) and *rap-1a* (**Supplementary Table 4** and **Figure 8A–C**) amino acid sequences using the ElliPro Server, which overlapped with the linear sequence of B-cell epitopes. The yellow color of the Ellipro graphs indicated the presence of these antigenic properties in that region of *sbp-2* (**Figure 7E**) and *rap-1a* (**Figure 8D**) correlating the BepiPred linear epitope prediction.

**Table 5:** Antigenicity of B-cell epitopes predicted from *sbp-2* and *rap-1a* amino acid sequences determined in the present study.

*B. bovis sbp-2* amino acid sequence				*B. bigemina rap-1a* amino acid sequence			
Epitopes^a^	Position		Conserved amino	Epitopes^a^	Position		Conserved amino
	Start	End	acid/total amino acid		Start	End	acid/total amino acid
LNPLVIK	42	48	7/7	TLLVTVSDYVHLPA	68	81	14/14
YYDVFLISPILNVDWL	77	92	10/16	EFIVNF	91	96	6/6
SLISSHSIRVHVDVVLQQFT	101	120	15/20	IMKHVSQPVKTA	104	115	12/12
LSLPLVVN	134	141	7/8	RNVVGQ	129	134	6/6
AHHLLDH	146	152	3/7				
EVIVVAN	184	190	7/7				

^a^Amino acid residues in the epitopes predicted from the deduced amino acid sequences of OK061181 (SBP-2) and OK061176 (RAP-1a) were analyzed for their diversity among Thailand sequences.

## Discussion

In Thailand, bovine babesiosis is a serious disease that causes clinical infection in cattle resulting in decreased livestock production and economic losses (Bock et al., 2004; Uilenberg, 2006; Simking et al., 2014). Currently, a molecular assay such as nested PCR is a sensitive diagnostic method and employed for detection and characterization of the parasites during the low level of parasitemia in infected animals (Smeenk et al., 2000; Altay et al., 2008; AbouLaila et al., 2010; Mosqueda et al., 2012). Our results showed a molecular detection of *Babesia* spp. in cattle blood sample in three regions (northern, western, and southern) of Thailand. The molecular assay revealed that of the cattle sampled, 2.58% (4/155) and 5.80% (9/155) were positive for *B. bovis sbp-2* and *B. bigemina rap-1a* genes, respectively. Both *sbp-2* and *rap-1a* genes have been employed as good markers for establishing phylogeographic patterns on the large scale and beneficial for epidemiological study of babesiosis (AbouLaila et al., 2010; Terkawi et al., 2011a; Cao et al., 2012; Moumouni et al., 2015; Zhou et al., 2016). The infection rate of *B. bovis* and *B. bigemina* in this study is lower than those reported by the epidemiology studies in Thailand that showed 5.3% (Simking et al., 2014) and 12.9% (Cao et al., 2012). In addition, the low occurrence of *B. bovis* compared to *B. bigemina* in the present study also agrees with previous reports (Awad et al., 2011; Terkawi et al., 2011a; Cao et al., 2012; Ibrahim et al., 2013; Simking et al., 2014; Jirapattharasate et al., 2016). Therefore, the chance of transmission of *B. bovis* is lower than that of *B. bigemina* in cattle in Thailand (Jirapattharasate et al., 2016). Moreover, the occurrence of *B. bovis* using *sbp-4* gene was found to be 11.2% in the northeast region of Thailand (Terkawi et al., 2011a), and the ITS1-5.8s rRNA gene has been used to determine the genetic diversity of *B. bigemina*-infected beef cattle in Thailand (Jirapattharasate et al., 2017). This is the first study that revealed a molecular detection of *Babesia* spp. in cattle in 5 provinces (Nan, Ratchaburi, Surat Thani, Nakhon Si Thammarat and Phatthalung) of Thailand. From our results, it is possible that the increase in livestock industry and urbanization might lead to migration of animals in these provinces and also a risk of transboundary disease might be increased. Additionally, the seasonal sampling and population density of ticks and cattle raising might be the risk factors for *Babesia* infections (Ahantarig et al., 2008; Iseki et al., 2010; Awad et al., 2011; Terkawi et al., 2011a; Simking et al., 2014).

The genetic diversity of *B. bovis* and *B. bigemina* strains based on the sequences of *sbp-2* and *rap-1a* genes, respectively, has been evaluated in several countries (Nagano et al., 2013; Moumouni et al., 2015; Guswanto et al., 2017; Herrera et al., 2017; Galon et al., 2019). Unfortunately, a little is known regarding the diversity of *B. bovis* and *B. bigemina* Thailand strains. In this work, the *sbp-2* and *rap-1a* genes in cattle population in the northern, western and southern regions of Thailand were used to assess the genetic diversity of *B. bovis* and *B. bigemina* in these areas, respectively. The phylogenetic analysis of *B. bovis sbp-2* gene Thailand strain showed 2 clades with other strains, while *B. bigemina rap-1a* gene Thailand isolate revealed a clade which showed phylogenetic proximity. Our bootstraps on the phylogenetic tree were 60-98% of bootstrap values, which are consistent with the of a majority-rule consensus tree of 1000 replicates for each alignment (Buckley and Cunningham, 2002; Soltis and Soltis, 2003). The results exhibited that the genetic diversity observed in the phylogram was confirmed by the high similarity values for *B. bovis sbp-2* gene (85.53-100%). This finding showed that the genetic diversity among *B. bovis* populations has been varied according to the geographical areas with further diversification resulting from local selection pressure. Furthermore, the high similarity value was detected for *B. bigemina rap-1a* gene (100%). This finding indicated the phylogenetic proximity of *B. bigemina rap-1a* gene circulating in both different countries and Thailand.

In this study, the genotype of *B. bovis sbp-2* and *B. bigemina rap-1a* sequences were established in the haplotype TCS networks. They were conducted with the sequences observed in this work together with other sequences obtained from GenBank database that was found in other regions of the world. Our finding revealed that there was a high genetic diversity of *B. bovis sbp-2* and *B. bigemina rap-1a* genes detected in the different haplotype networks in Thailand and worldwide when compared to the earlier study of Mendes and colleagues (2019) who showed the diversity of haplotypes of *B. bovis msa-2b* gene (1.000 ± 0.027) and *B. bovis msa-2c* gene (0.9794 ± 0.039) detected in Brazilian cattle (Mendes et al., 2019). In addition, sequences observed in this work were clustered with sequences from different countries, indicating that there has been no unique haplotype in Thailand. These two sequences shared genetic traits with all sequences previously investigated worldwide. These indicated that the genetic diversity among *Babesia* populations varied in accordance with the geographical areas (Homer et al., 2000; Bock et al., 2004; Uilenberg, 2006). Notably, it is possible that the *B. bovis* and *B. bigemina* strains acquainted by importing cattle from other countries might have contributed to the observed genetic diversity of *sbp-2* and *rap-1a* gene sequences in Thailand (Iseki et al., 2010; Lau et al., 2010; Tattiyapong et al., 2016; Sivakumar et al., 2018).

Regarding the analysis of *Babesia* nucleic and amino acid sequences, the *sbp-2* genes have higher levels of sequence polymorphism than those of *rap-1a* genes. For codon degeneration, most codon degeneracies of *B. bovis sbp-2* gene showed the synonymous (K_s_) and non-synonymous (K_n_) substitutions values were 0.1875 and 0.0708, respectively, while the *B. bigemina rap-1a* gene showed the K_s_ and K_n_ values were 0.0129 and 0.0064, respectively. The non-synonymous to synonymous mutations ratio (K_n_/K_s_) of *B. bovis sbp-2* and *B. bigemina rap-1a* genes were 0.3776 and 0.4961 which interpreted as neutral selection (Niu et al., 2015; Rittipornlertrak et al., 2017). This indicated that the evolutionary substitution of mutations in coding sequences might not affect the protein sequence modification and could not impact change the biological function effect of a protein (Goymer, 2007; Choi and Chan, 2015).

In this study, our results exhibited the polymorphism with 104 entropy peaks reaching up to 1.02809 for *B. bovis sbp-2* gene sequences and with 7 entropy peaks reaching up to 0.56234 for *B. bigemina rap-1a* gene sequences. In addition, the analysis of amino acid sequences of *B. bovis sbp-2* and *B. bigemina rap-1a* sequences showed the polymorphism with 46 and 4 entropy peaks continuing up to 1.2064 and 0.56234, respectively. These findings indicated that different genotypes might involve being a genetic diversity of *Babesia* distribution in Thailand. Notably, our results were in line with the previous report of Mendes et al. (2019) who exhibited the entropy values of *B. bovis msa-2b* gene reaching up to 1.53, and *B. bovis msa-2c* gene with entropy values reaching up to 1.09. The high entropy values indicate a high number of variations of single-nucleotide polymorphisms (SNP), which is associated with a high number of nucleotide and amino acid variables (Mendes et al., 2019). The low entropy values indicate a few numbers of variations of SNP in each sequence (Pham, 2010). Furthermore, motif analysis gains information for determining the functional properties of biopolymers (nucleotide and protein subunits) and representing the roles in biological networks, such as DNA binding sites and protein interaction domains (Bailey et al., 2009; Nystrom and McKay, 2021). The analysis of the conserved motifs was correlated with our findings, and these motifs were essential for the parasite in evading the host immune system (Dijkstra et al., 2018).

In the present study, the results showed that the predicted B-cell epitopes of *sbp-2* and *rap-1a* amino acid sequences among Thailand isolates were relatively conserved. Although many epitopes of *sbp-2* and *rap-1a* amino acid sequences were conserved as functional peptides, they are not located within the signal peptide. In addition, the *sbp-2* and *rap-1* proteins were identified as the conserved peptide containing B-cell epitopes in *B. bovis* that involved Th_1_ immune response in an *in vivo* study (Gallego-Lopez et al., 2019; Hidalgo-Ruiz et al., 2022). However, further research into the functional properties of the *rap-1a* protein in *B. bigemina* was required. The high polymorphism investigated among the epitope prediction of *sbp-2* amino acid sequences might exhibit the highest evolutionarily variability, suggesting that the antigenicity of variable sequence and evolutionarily conserved regions could induce immunological tolerance (Benjamin et al., 1984). Furthermore, *B. bovis sbp-2* protein has been associated to increased parasite virulence and severe disease outcomes (Gallego-Lopez et al., 2019). This implies that the *sbp-2* protein may help parasites to evade the host defense mechanism, and it is believed that *B. bovis* infection is a severe disease because of this. (Suarez et al., 2000; Bock et al., 2004; Yusuf, 2017; Gallego-Lopez et al., 2019).

## Conclusions

The present study importantly indicates a molecular detection and genetic diversity of *B. bovis* and *B. bigemina* in cattle samples from Thailand’s northern, western, and southern regions. Our results revealed that the diversity of *B. bovis spb-2* gene is genetically diverse, whereas that of *B. bigemina rap-1a* gene is conserved in Thailand and worldwide. These findings could be used to improve the insight of molecular phylogenetics and diversity among *sbp-2* of *B. bovis* and *rap-1a* of *B. bigemina* Thailand strains. Therefore, it is possible that our findings will be useful in development of immunodiagnostic tests and vaccine strategies. However, we still encourage further *in vivo* validation studies for further prevention and control of bovine babesiosis throughout the country in order to mitigate the infection of cattle vector-borne parasites.

## Data availability statement

The datasets presented in this study can be found in online repositories. The names of the repository/repositories and accession number(s) can be found in the article/**Supplementary Material**.

## Ethics statement

All experimental procedures involving animals were approved by the Animal Care and Use Committee (IMBMU-ACUC), Institute of Molecular Biosciences, Mahidol University, Thailand. All suitable international, national and/or institutional guidelines for animal care and use were followed. As well, we have received consent to collect the cattle blood sample at the animal farm.

## Author contributions

NS: Conceptualization, Methodology, Investigation, Writing original draft version, Data Curation, Visualization and Project administration. PN: Visualization and Resources. NP: Visualization and Resources. SM: Resources. AW: Resources. WJ: Resources. SS: Resources. RC: Resources. PA: Conceptualization, Investigation, Methodology, Visualization, Data Curation, Project administration, Validation, Writing-Review & Editing, Supervision, Resources and Funding acquisition. All authors contributed to the article and approved the submitted version.

## Funding

This research paper is supported by Specific League Funds and Basic Research Fund: fiscal year 2022 from Mahidol University to PA.

## Acknowledgments

We are also grateful to Surat Thani artificial insemination and biotechnology center, Surat Thani province, Thailand and Mrs. Pacharaporn Khumpim for their providing some cattle blood samples. Likewise, we would like to thank Prof. Dr. Peter Quantick, Research Director, RedKnight Consultancy Ltd, UK for assistance in correcting the manuscript.

## Conflict of interest

The authors declare that the research was conducted in the absence of any commercial or financial relationships that could be construed as a potential conflict of interest.

## Publisher’s note

All claims expressed in this article are solely those of the authors and do not necessarily represent those of their affiliated organizations, or those of the publisher, the editors and the reviewers. Any product that may be evaluated in this article, or claim that may be made by its manufacturer, is not guaranteed or endorsed by the publisher.

## Supplementary material

Supplementary Figure 1 PCR products of *B. bovis sbp-2* and *B. bigemina rap-1a* genes of Thailand strain show a 584 bp fragment of *sbp-2* gene **(A)** from Muang (lane 1-2), Don-Sak (lane 3) and Pa-phayom (lane 4) districts, and a 412 bp fragment of *rap-1a* gene **(B)** from Muang (lane 1), Photharam (lane 2), Don-sak (lane 3), Cha-wang (lane 4) and Pa-phayom (lane 5) districts. Lanes N and P indicate negative and positive controls, respectively. The molecular size standard (M) is a 100-bp ladder.

Supplementary Figure 2 Multiple sequence alignment of the *sbp-2* gene sequences among *B. bovis* different strains (visualized with JalView). The sequences obtained in the present study are shown in boldface latter. The green letter indicates identical consensus. The red rectangle indicates non-consensus.

Supplementary Figure 3 Multiple sequence alignment of the *rap-1a* gene sequences among *B. bigemina* different strains (visualized with JalView). The sequences detected in the present study are exhibited in boldface latter. The green letter represents identical consensus. The red rectangle represents non-consensus.

Supplementary Figure 4 Alignment of amino acid sequences of *B. bovis sbp-2* **(A)** and *B. bigemina rap-1a* **(B)** from Thailand strain (boldface letter) and other strains (visualized with JalView). The identical residues of SBP-2 and RAP-1a are shown in yellow letters. The red rectangle indicates non-conserved residues.
