# Supplementary figures and images for: Molecular characterization and genetic diversity of Babesia bovis and Babesia bigemina of cattle in Thailand
Source: Front Cell Infect Microbiol. 2022 Nov 29;12:1065963. doi: 10.3389/fcimb.2022.1065963 (PMC9744959; doi:10.3389/fcimb.2022.1065963)

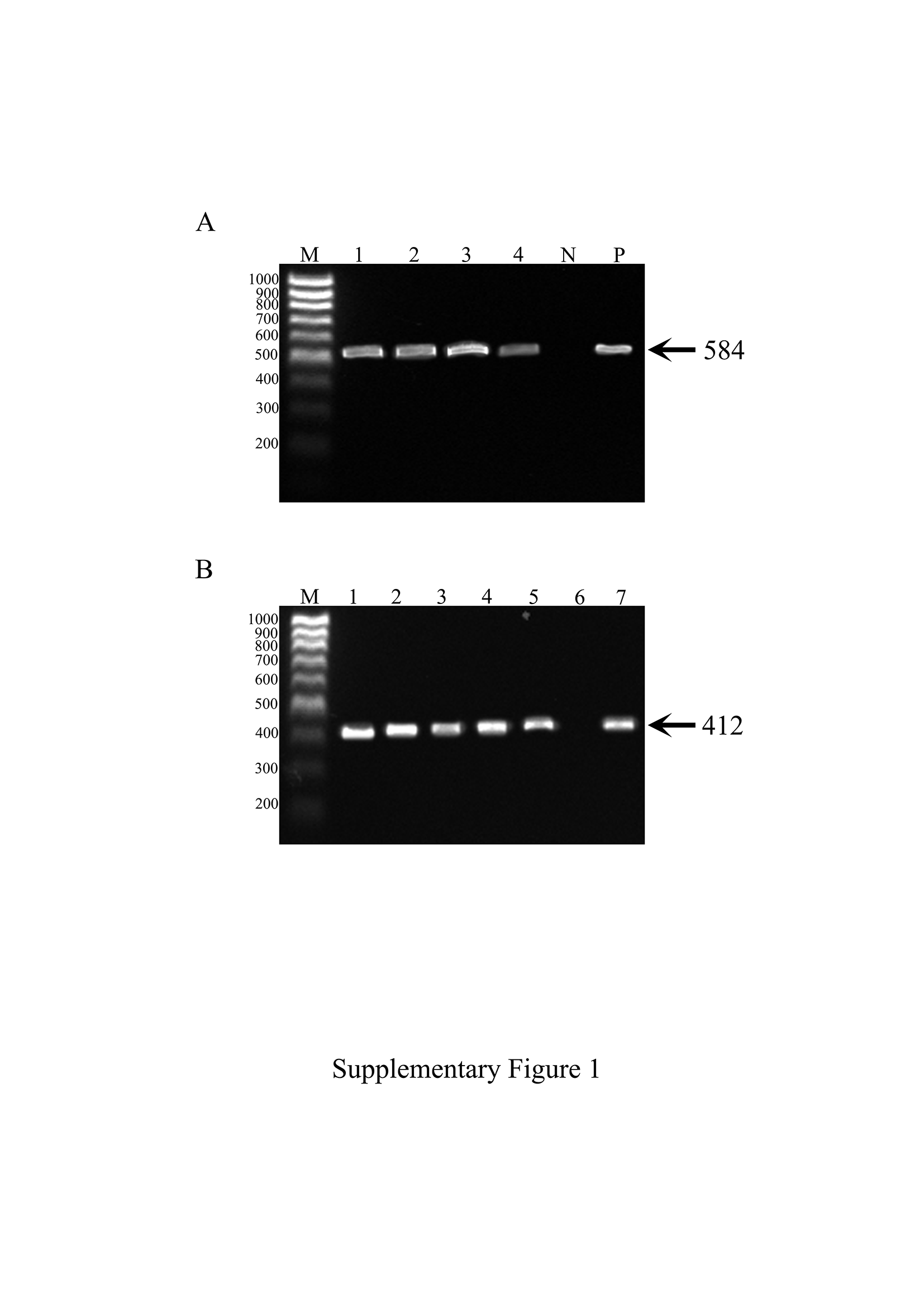

Supplement: Supplementary Figure 1 — PCR products of B. bovis sbp-2 and B. bigemina rap-1a genes of Thailand strain show a 584 bp fragment of sbp-2 gene (A) from Muang (lane 1-2), Don-Sak (lane 3) and Pa-phayom (lane 4) districts, and a 412 bp fragment of rap-1a gene (B) from Muang (lane 1), Photharam (lane 2), Don-sak (lane 3), Cha-wang (lane 4) and Pa-phayom (lane 5) districts. Lanes N and P indicate negative and positive controls, respectively. The molecular size standard (M) is a 100-bp ladder. [file DataSheet_1.zip › Supplementary Material/Supp Fig 1.tif]

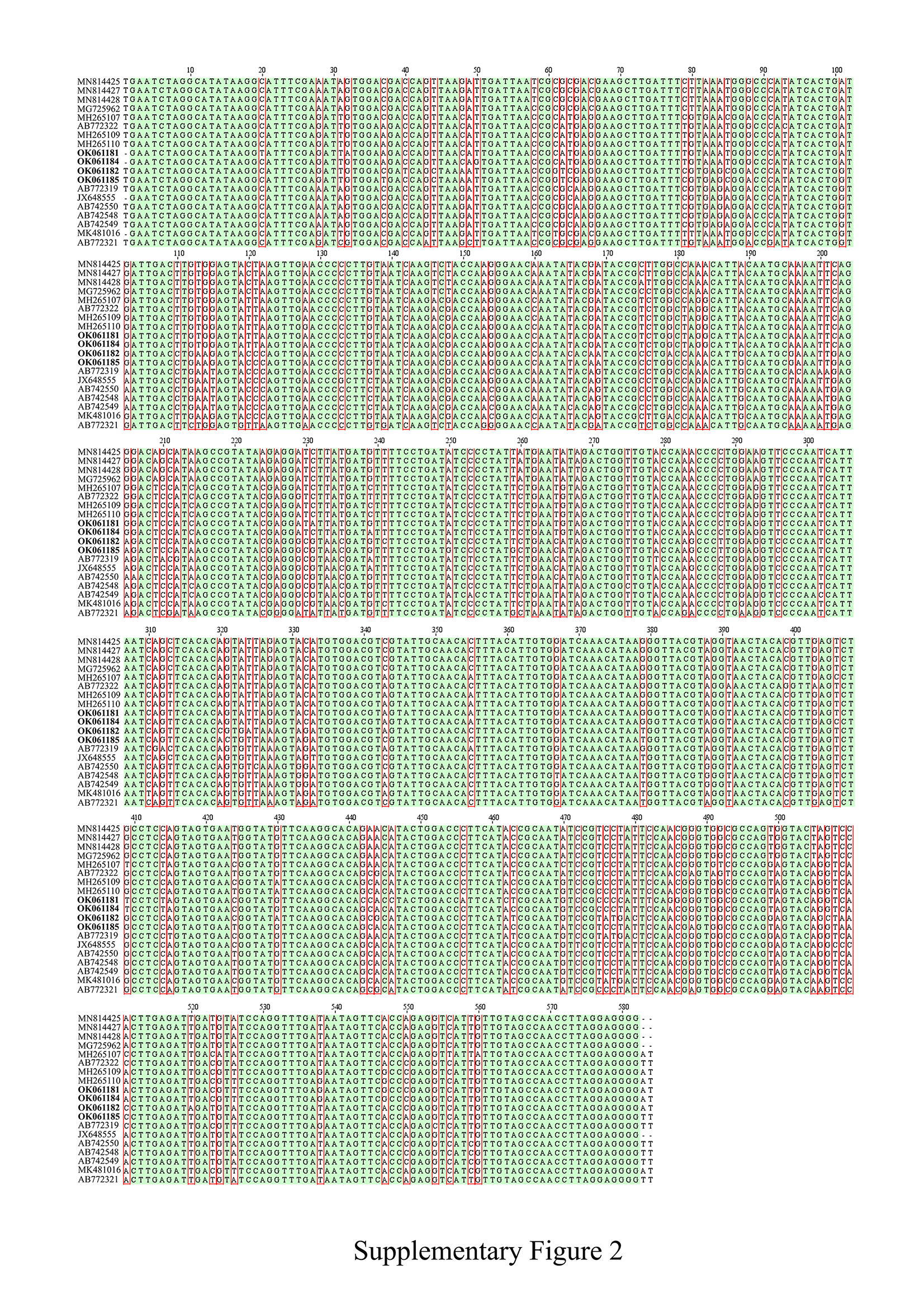

Supplement: Supplementary Figure 1 — PCR products of B. bovis sbp-2 and B. bigemina rap-1a genes of Thailand strain show a 584 bp fragment of sbp-2 gene (A) from Muang (lane 1-2), Don-Sak (lane 3) and Pa-phayom (lane 4) districts, and a 412 bp fragment of rap-1a gene (B) from Muang (lane 1), Photharam (lane 2), Don-sak (lane 3), Cha-wang (lane 4) and Pa-phayom (lane 5) districts. Lanes N and P indicate negative and positive controls, respectively. The molecular size standard (M) is a 100-bp ladder. [file DataSheet_1.zip › Supplementary Material/Supp Fig 2.tif]

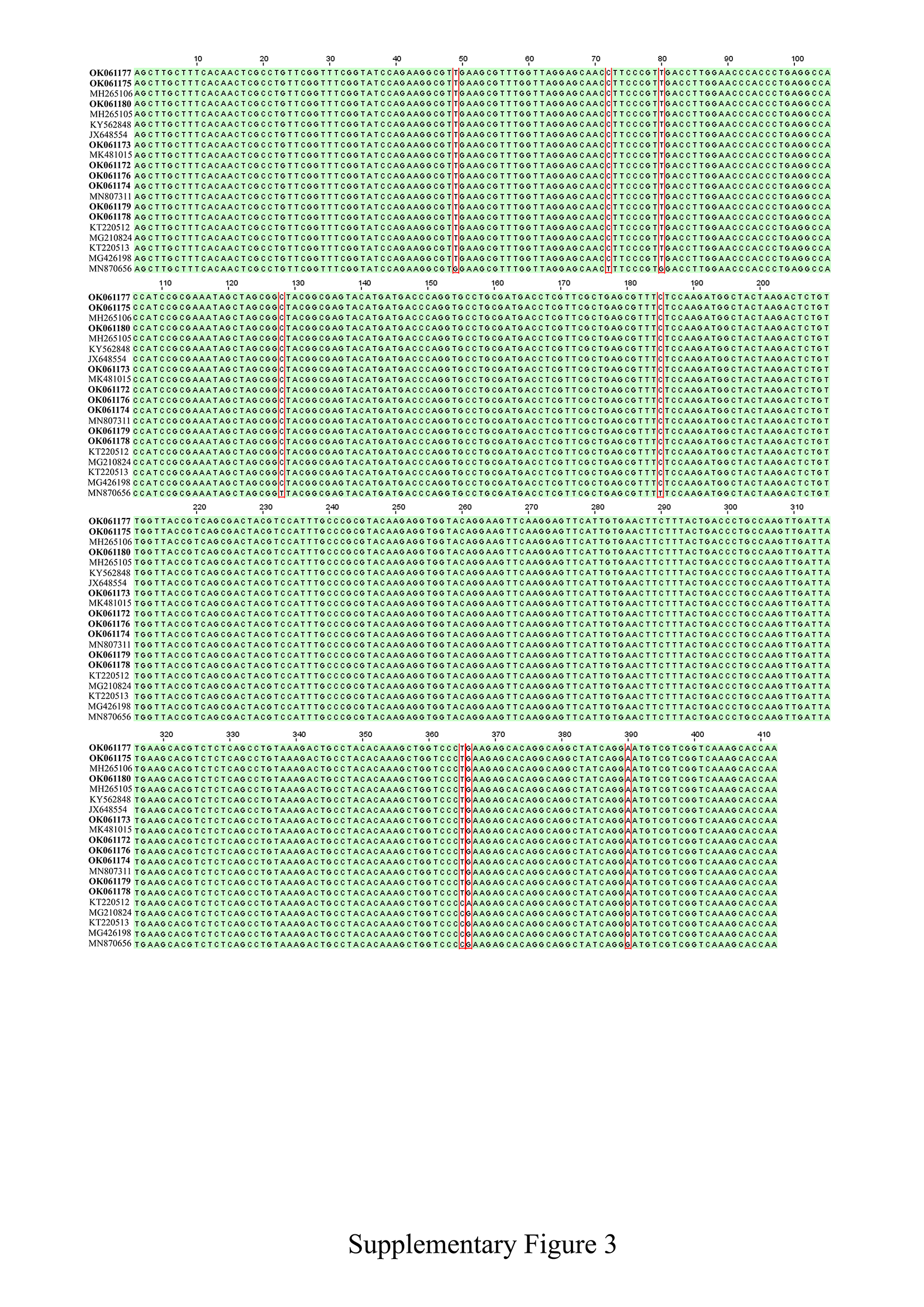

Supplement: Supplementary Figure 1 — PCR products of B. bovis sbp-2 and B. bigemina rap-1a genes of Thailand strain show a 584 bp fragment of sbp-2 gene (A) from Muang (lane 1-2), Don-Sak (lane 3) and Pa-phayom (lane 4) districts, and a 412 bp fragment of rap-1a gene (B) from Muang (lane 1), Photharam (lane 2), Don-sak (lane 3), Cha-wang (lane 4) and Pa-phayom (lane 5) districts. Lanes N and P indicate negative and positive controls, respectively. The molecular size standard (M) is a 100-bp ladder. [file DataSheet_1.zip › Supplementary Material/Supp Fig 3.tif]

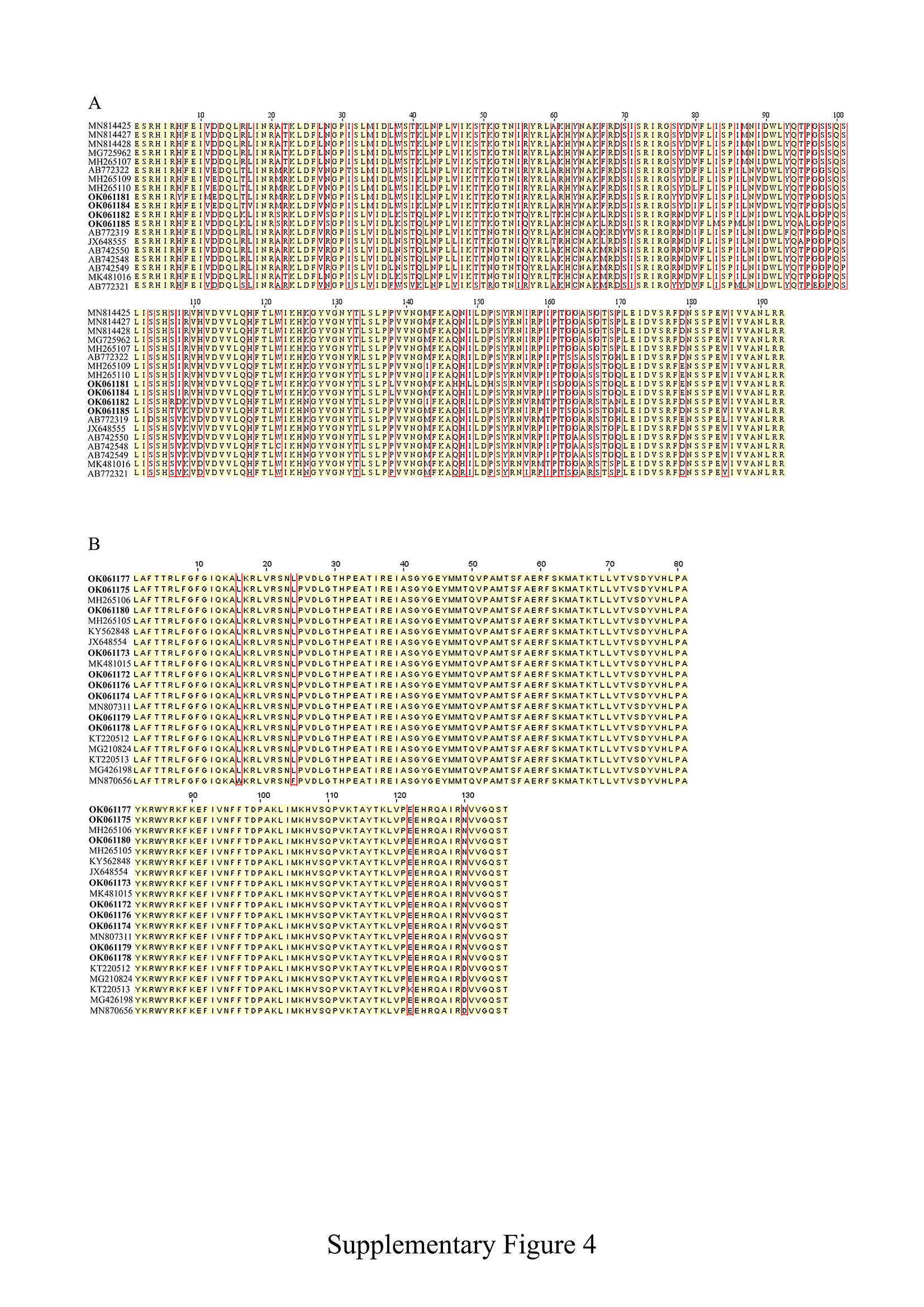

Supplement: Supplementary Figure 1 — PCR products of B. bovis sbp-2 and B. bigemina rap-1a genes of Thailand strain show a 584 bp fragment of sbp-2 gene (A) from Muang (lane 1-2), Don-Sak (lane 3) and Pa-phayom (lane 4) districts, and a 412 bp fragment of rap-1a gene (B) from Muang (lane 1), Photharam (lane 2), Don-sak (lane 3), Cha-wang (lane 4) and Pa-phayom (lane 5) districts. Lanes N and P indicate negative and positive controls, respectively. The molecular size standard (M) is a 100-bp ladder. [file DataSheet_1.zip › Supplementary Material/Supp Fig 4.tif]
